# Reassessing *Stephanofilaria stilesi* dermatitis in cattle, with characterization of molecular markers for confirming diagnosis

**DOI:** 10.1186/s13071-023-05905-y

**Published:** 2023-08-12

**Authors:** Clinson C. Lui, Matthew Kulpa, Guilherme G. Verocai, Aníbal G. Armién, Erin E. Edwards, Dominique J. Wiener, Raquel R. Rech

**Affiliations:** 1 Department of Veterinary Pathobiology, School of Veterinary Medicine and Biomedical Sciences, Texas A&M University, College Station, TX USA; 2 California Animal Health & Food Safety Laboratory System, Davis, CA USA; 3 Texas A&M Veterinary Medical Diagnostic Laboratory, College Station, TX USA

**Keywords:** Filariidae, Filarial dermatitis, Stephanofilariasis, Thelazioidea, Transmission electron microscopy (TEM), Vector-borne diseases

## Abstract

**Background:**

*Stephanofilaria stilesi* is a vector-borne filarioid nematode of cattle in North America that is transmitted via the hematophagous horn fly (*Haematobia irritans*) intermediate host. Despite being relatively common, little attention has been given to a thorough description of *S. stilesi* lesions and the potential integration of pathological and molecular diagnostic findings to confirm infection.

**Methods:**

To characterize the cutaneous lesions caused by *S. stilesi* in cattle (*Bos taurus taurus* and *Bos taurus indicus*), skin of the ventral abdominal midline was collected from 22 animals during postmortem examination. Skin samples were processed for histology, transmission electron microscopy (TEM), DNA extraction, PCR, and Sanger sequencing targeting molecular markers cytochrome oxidase c subunit 1 (*cox1*), 12S, 18S rDNA, and 28S rDNA.

**Results:**

Macroscopically, lesions ranged from 5 × 4 cm to 36 × 10 cm, consisting of one large single lesion, or two to four ovoid areas at the ventral abdominal midline, surrounding the umbilicus. Each lesion presented as ulcerative dermatitis with dry, serocellular crusts, or alopecic and lichenified areas. Histologically, eosinophilic, neutrophilic, and ulcerative dermatitis with furunculosis, folliculitis, and epidermal hyperplasia was observed. Cross sections of adult nematodes were identified in ~ 60% of the cases (*n* = 13) within intact follicles, sebaceous ducts, crusts, and areas of furunculosis. *Stephanofilaria* first-stage larvae (L1) were observed in five cases within “vitelline membranes” in the superficial dermis and crusts. Ultrastructurally, the L1 cross sections were compounded of smooth multilayered cuticle and somatic cells. The “vitelline membrane” is a tri-layered membrane where L1 are suspended in a matrix. *Stephanofilaria stilesi* DNA was found in 5 out of the 13 cases in which adults or L1 were histologically observed (38%) and in 1 out of the 9 cases without adults or L1 present (11%). Phylogenetic analyses suggest a closer relationship of the genus *Stephanofilaria* with Thelazioidea, instead of the family Filariidae (Filarioidea), in which it has been historically allocated.

**Conclusions:**

Our study improved the characterization of lesions and described ultrastructural findings of *S. stilesi* and highlights that molecular tools should be utilized in combination with histology for improved diagnostic resolution.

**Graphical Abstract:**

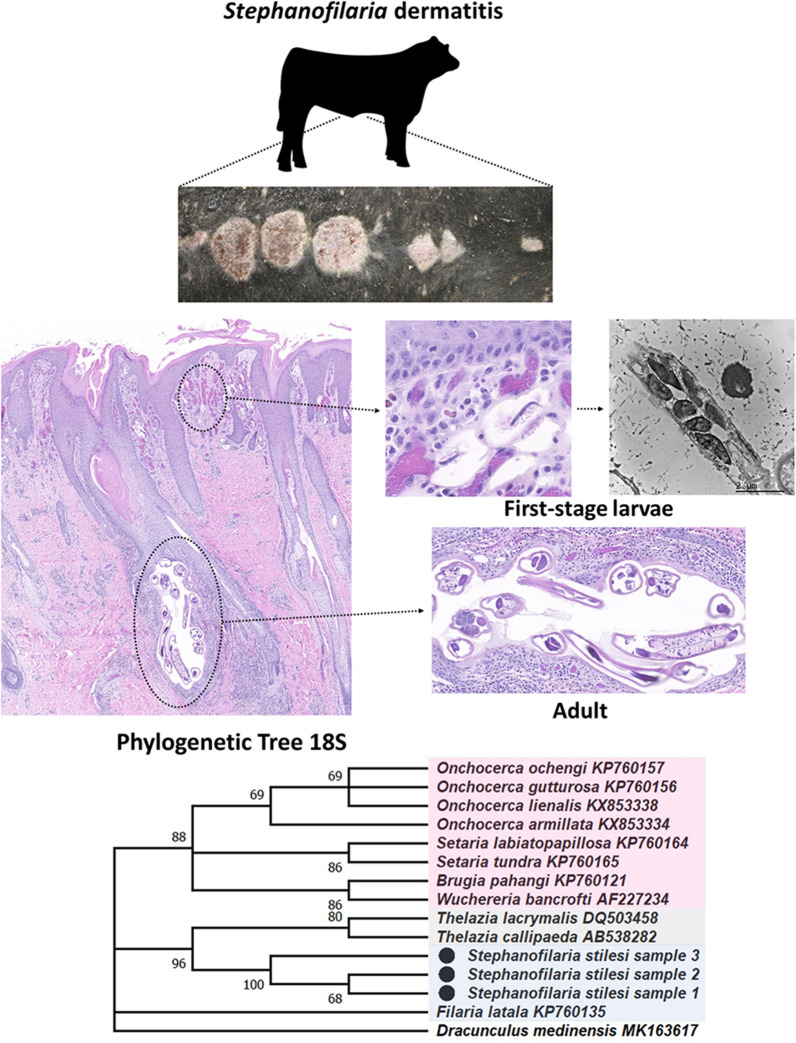

## Background

*Stephanofilaria stilesi* Chitwood, 1934, is a vector-borne filarioid nematode parasite of cattle in North America, which seem to be more prevalent in the southern and western USA and has also been described in western Canada [1, 2]. The horn fly, *Haematobia irritans*, the intermediate host of *S. stilesi*, is also among the most common and economically important ectoparasites of cattle in North America and worldwide [3–5]. Horn flies are infected with *S. stilesi* first-stage larvae after blood-feeding on skin lesions associated with this nematode generally located along the ventral midline of the hosts. These first-stage larvae (L1) develop to the infective third-stage larvae (L3) during a 2- to 3-week period within the horn fly. When the fly feeds again on a susceptible host, the L3s are inoculated into the skin of the cattle, and eosinophilic crusting dermatitis develops from the nematodes over 2 weeks. Nematodes will then develop into adults and sexually reproduce to form more L1 [1]. While most literature to date refers to *S. stilesi* L1 as microfilariae [1, 2], we opted to adjust this terminology based on morphological and phylogenetic evidence from recent and our present work.

Stephanofilariasis, caused by different species within the genus *Stephanofilaria* Ihle & Ihle-Landernberg, 1933, is a common skin lesion found in cattle from North American and other areas of the world, including India, Australia, and Brazil [1, 2, 6, 7]. The condition will often present as a circumscribed crusting dermatitis along the ventral midline of the body between the sternum and umbilicus but can affect other areas including the ears, neck, withers, hump, back, and scrotum [8, 9]. However, stephanofilariasis has been reported to cause more severe skin lesions in host species other than cattle, including pigs, rhinoceros, giraffes, and hippopotamuses [10–14]. In addition to its dermatological impact, clinical or subclinical impacts of *H. irritans* with or without *S. stilesi* on cattle health and production may include effects on average daily weight gain, hide quality, and feed intake [4, 15, 16]. Microscopically, adult nematodes are usually present within hair follicles, sebaceous ducts, and crusts. Secondary furunculosis and folliculitis along with epidermal hyperplasia, ulceration, and erosion are common. First-stage larvae are contained in a “vitelline membrane” that is free within the superficial dermis and incites a perivascular dermatitis [15].

Despite being a common infection among North American cattle, *S. stilesi* remains an understudied parasitic nematode. In fact, much of the scientific literature appears dated regarding the gross and histopathological description of stephanofilariasis [17–19]. Moreover, *S. stilesi* has never been molecularly characterized, to the best of our understanding. This has impaired an informed assessment of its taxonomic placement within the family Filariidae and superfamily Filarioidea, which has been questioned by early researchers based on its biology and certain morphological features. Our goal was to fill these gaps in knowledge and provide an improved *S. stilesi* diagnosis through a combination of molecular, transmission electron microscopy, and histopathological analyses.

## Methods

### Sample collection and histopathology

Twenty-two skin samples with characteristic lesions of stephanofilariasis were collected between June 2020 and May 2021 from cattle in Texas, USA. The skin samples were obtained through the necropsy service at Texas A&M University and Texas A&M Veterinary Medical Diagnostic Laboratory (TVMDL). The animals used in this study were presented for unrelated causes of death with no primary complaint for dermatological lesions. Characteristic lesions of stephanofilariasis were identified incidentally and collected for evaluation. After retrieval of skin lesions, thin, 2–6-cm-long samples were obtained throughout affected areas and placed in 10% buffered formalin. After 1–2 days of fixation, histology and hematoxylin and eosin (H&E) staining was performed through routine processing. The remainder of each skin sample was stored at − 80 °C for subsequent molecular screening.

### Transmission electron microscopy

Formalin-fixed, paraffin-embedded (FFPE) skin tissue with L1 was subjected to transmission electron microscopy (TEM). Tissue blocks were deparaffinized in a decreasing alcohol gradient and then postfixed in Karnovsky solution followed by a second postfixation with 1% osmium tetroxide in 0.1 M sodium cacodylate buffer (Electron Microscopy Sciences, Hatfield, PA). Afterward, samples were routinely processed and embedded in Embed 810 resin (Electron Microscopy Sciences) as described in Armién et al. [20]. Tissue blocks were trimmed and sectioned on a Leica UC6 ultramicrotome (Leica Microsystems, Vienna, Austria). Contrasted thin sections (60–70 nm) were visualized using a JEOL 1400 transmission electron microscope (JEOL LTD, Tokyo, Japan). Images focused on the vitelline membrane and L1 were obtained and analyzed using a OneView camera system Model 1095, 16 megapixels, with the Gatan Microscope Suite (GMS3.0) (Gatan Inc, Pleasanton, CA, USA).

### Dissection and DNA extraction

Skin samples (*n* = 22), frozen at − 80 °C, with confirmed histological evidence of adult nematodes, were thawed at room temperature. After 24 h, 0.5 cm^3^ of skin immediately ventral to the epidermis was dissected from each sample at the characteristic lesion. Genomic DNA extraction was performed utilizing a Qiagen DNeasy^©^ Blood & Tissue kit (Qiagen, Valencia, CA, USA) with ATL buffer and proteinase K. Tissue samples were incubated in a mixing heat block overnight at 56 °C and then centrifuged for a minute at 8000×*g*. The remaining protocol steps followed the manufacturer’s instructions. DNA lysates were kept refrigerated at − 20 °C until further processing.

### Molecular characterization, diagnostic screening, and sequencing

Multiple genetic markers widely used for diagnostic purposes and phylogenetic studies on filarioid nematodes were initially targeted, including three mitochondrial genes: 12S, NADH dehydrogenase subunit 5 (*nd5*), and cytochrome oxidase c subunit 1 (*cox1*); two ribosomal genes: 18S rDNA and 28S rDNA; and three nuclear genes: myosin heavy chain (*MyoHC*), RNA polymerase II large subunit (*rbp1*), and 70 kilodalton heat-shock protein (*hsp70*) (Table 1) [45]. Clinical cases confirmed to be positive for nematodes on histopathology were utilized for molecular characterization. After this initial step, 22 cases exhibiting characteristic *Stephanofilaria* sp. lesions were screened for nematode DNA with appropriate primers (Table 2). DNA extraction from three skin pieces and PCR were performed in triplicates on skin tissue dissections, and the protocols along with polymerase chain reaction annealing temperatures followed a previous study. However, in the event the initial cycling conditions were unsuccessful, a PCR gradient was performed to determine optimal annealing temperatures for subsequent reactions (Table 1) [45]. Potential PCR products were subjected to 1% agarose gel stained with SYBR® Safe DNA Gel Stain (Thermo Fisher Scientific, USA) and visualized in UV light to determine whether amplicons were present. All amplification reactions were performed in 25 μl volumes that included 0.625 μM of each primer, 1 × GoTaq® Green Master Mix (Promega Corporation, Madison, WI, USA), and 2.5 μl DNA template. If amplicons were present, a Cycle Pure ENZA kit (Omega Bio-Tek, Norcross, GA, USA) was used to purify DNA using the manufacturer’s protocol. Products were then submitted for Sanger sequencing to confirm the presence of *S. stilesi*.

**Table 1:** Primers utilized to amplify *Stephanofilaria stilesi* DNA along with annealing temperatures and average base pair sizes

Primer	Sequences	Size (base pairs)	Tm ℃	Successful PCR	References
*cox1*	Forward: 5ʹ-TGA TTG GTG GTT TTG GTA A-3ʹ	~ 700 bp	52 °C	Yes	Casiraghi et al. [44]
	Reverse: 5ʹ-ATA AGT ACG AGT ATC AAT ATC-3ʹ				
*12S rDNA*	Forward: 5ʹ-TCG GCT ATG CGT TTT AAT TTT-3ʹ	~ 533 bp	50 °C	Yes	Casiraghi et al. [44]
	Reverse: 5ʹ-CAA CTT ACG CCC CTT TAG GC-3ʹ				
*18S rDNA*	Forward: 5ʹ-ACC GCC CTA GTT CTG ACC GTA AA-3ʹ	~ 740 bp	58 °C	Yes	Lefoulon et al. [45]
	Reverse: 5ʹ-GGT TCA AGC CAC TGC GAT TAA AGC-3ʹ				
*28S rDNA*	Forward: 5ʹ-CCTCAACTCAGTCGTGATTACC-3ʹ	~ 1150 bp	61.5 °C	Yes	Lefoulon et al. [45]
	Reverse: 5ʹ-CTCTGGCTTCATCCTGCTCA-3ʹ				
*MyoHC*	Forward: 5ʹ-GCA TCA RGA AGA AAT TAA TCG-3ʹ	~ 785 bp	59 °C	No	Lefoulon et al. [45]
	Reverse: 5ʹ-GCT TCA ATT TCY TCC TCC AT-3ʹ				
*rbp1*	Forward: 5ʹ-ACT GCA AAY ACW GCW ATT TA-3ʹ	~ 640 bp	53 °C	No	Lefoulon et al. [45]
	Reverse: 5ʹ-ACR TGA TTC ATT TCR CGT TC-3ʹ				
*hsp70*	Forward: 5ʹ-TCR GAT TTC TTY TCT GGY A-3ʹ	~ 610 bp	55 °C	No	Lefoulon et al. [45]
	Reverse: 5ʹ-GTY TGY TTC ATA TTG AAY GC-3ʹ				
*nad5*	Forward: 5ʹ-TTG GTT GCC TAA GGC TAT GG-3ʹ	~ 470 bp	50 °C	No	Morales-Hojas et al. [46]
	Reverse: 5ʹ-CCC CTA GTA AAC AAC AAA CCA CA-3ʹ

**Table 2:** Retrospective stephanofilariasis case screening with *cox1* primers

*Stephanofilaria* histology cases	Total cases (*N* = 22)	Triplicates (*N* = 66)	Cases with *Stephanofilaria stilesi* sequence	Percentage	All replicates with *Stephanofilaria stilesi* sequence	Percentage
Adult worm present	13	39	5/13	38	7/39	18
Adult worm absent	9	27	1/9	11	1/27	4
L1 present	5	15	3/5	60	4/15	27
L1 absent	17	51	3/17	18	4/51	8
Adult + L1 present	5	15	3/5	60	4/15	27
Adult + L1 absent	9	27	1/9	11	1/27	4
Adult or L1 present	8	24	2/8	25	3/24	13

Cases were considered microscopically positive or negative depending on finding of adult worm or first-stage larvae (L1). Three random tissue samples were dissected from each case at the characteristic lesions caused by *S. stilesi* and subjected to DNA extraction, PCR with *cox1* primers, gel electrophoresis, and Sanger sequencing

### Phylogenetic analyses

Sequences were aligned and edited using MEGA X software [21]. Phylogenetic trees of the partial *cox1* gene (681 bp) were constructed by utilizing the maximum likelihood method and Tamura–Nei model with gamma distribution in 2000 bootstrap replicates. Sequences at the *cox1* gene for related nematodes within the families Filariidae and Onchocercidae were included in phylogenetic analyses. *Dracunculus medinensis* (Linnaeus, 1758) was used as an outgroup outside the superfamily Filarioidea but within the suborder Spirurina as well as available sequences of species within the genus *Thelazia*. Phylogenetic trees were also generated for 12S using a Hasegawa-Kishino-Yano model with gamma distribution, 18S rDNA using a Kimura 2-parameter model with gamma distribution, and 28s rDNA using a General Time Reversible model with a gamma distribution in 2000 bootstrap replicates (Fig. 4).

## Results

### Clinical and gross findings

**Fig. 1:**
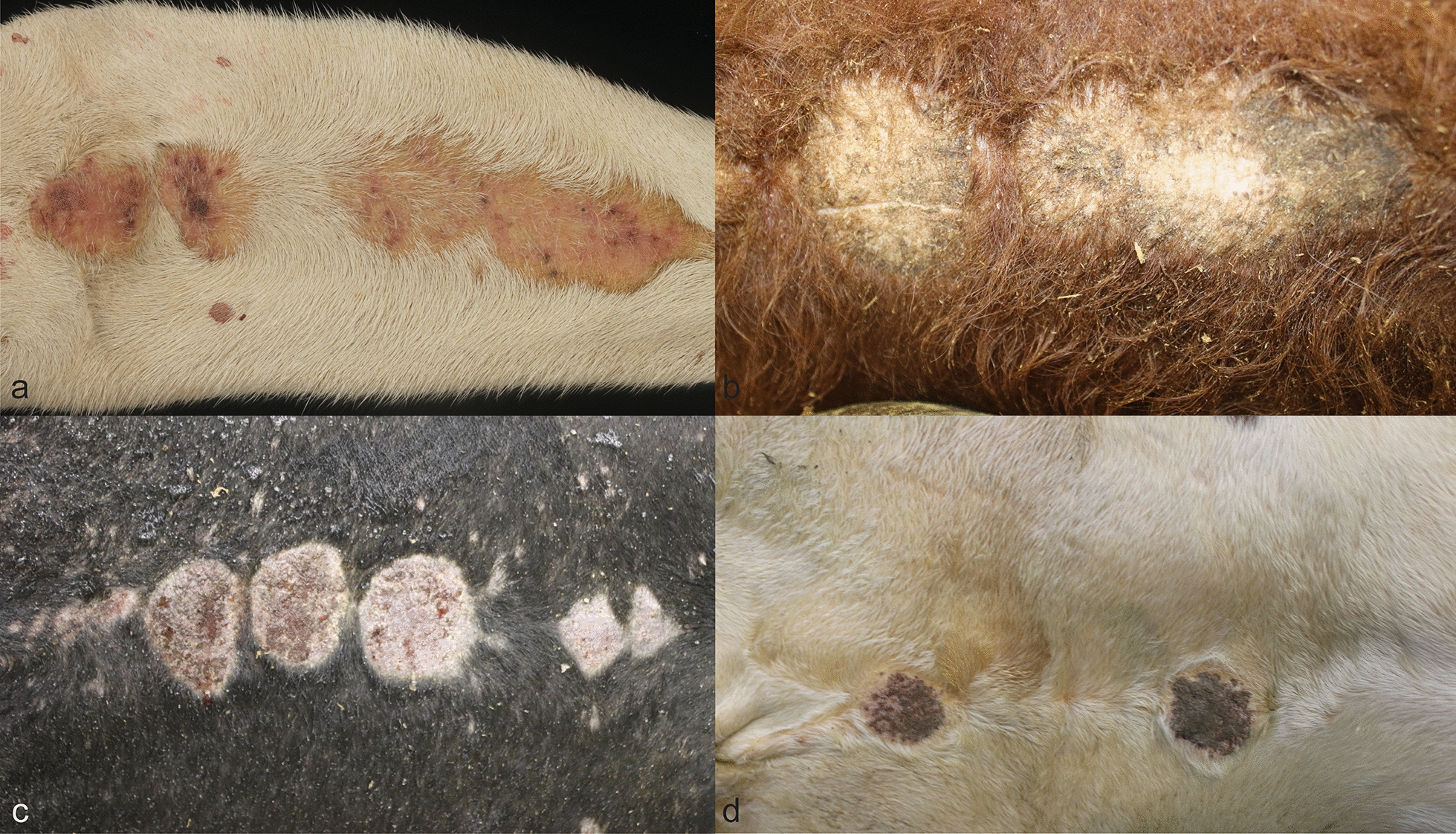
Characteristic lesions of *S. stilesi* dermatitis on the ventral abdomen of cattle of different ages and breeds. Skin lesions are characterized by multiple, round, 3 to 8 cm in diameter, alopecic, crusted areas. **a** 6-year-old Zebu cow. **b** 1-year-old Hereford heifer. **c** 4-year-old black Angus bull. **d** 1-year-old Hereford bull

The breeds of cattle with typical stephanofilariasis consisted of seven Hereford, seven Black Angus or Brangus, one Longhorn, one Maine-Anjou, two Charolais, one Beefmaster, one Jersey, one Holstein, and one unknown. The cattle ranged from 8-month-old to 12-year-old and 17 were female. Lesions ranged from 5 × 4 cm to 36 × 10 cm and were presented as one large single lesion or three to four ovoid areas at the ventral abdominal midline, surrounding the umbilicus. Each lesion presented as an ulcerative dermatitis with dry serocellular crusts or alopecic and lichenified areas (Fig. 1). Gross lesions associated with stephanofilariasis were evenly distributed throughout the time examined, with no overt seasonality. Severity of lesions ranged from moderate to severe, with no change in severity in any given month.

**Fig. 2:**
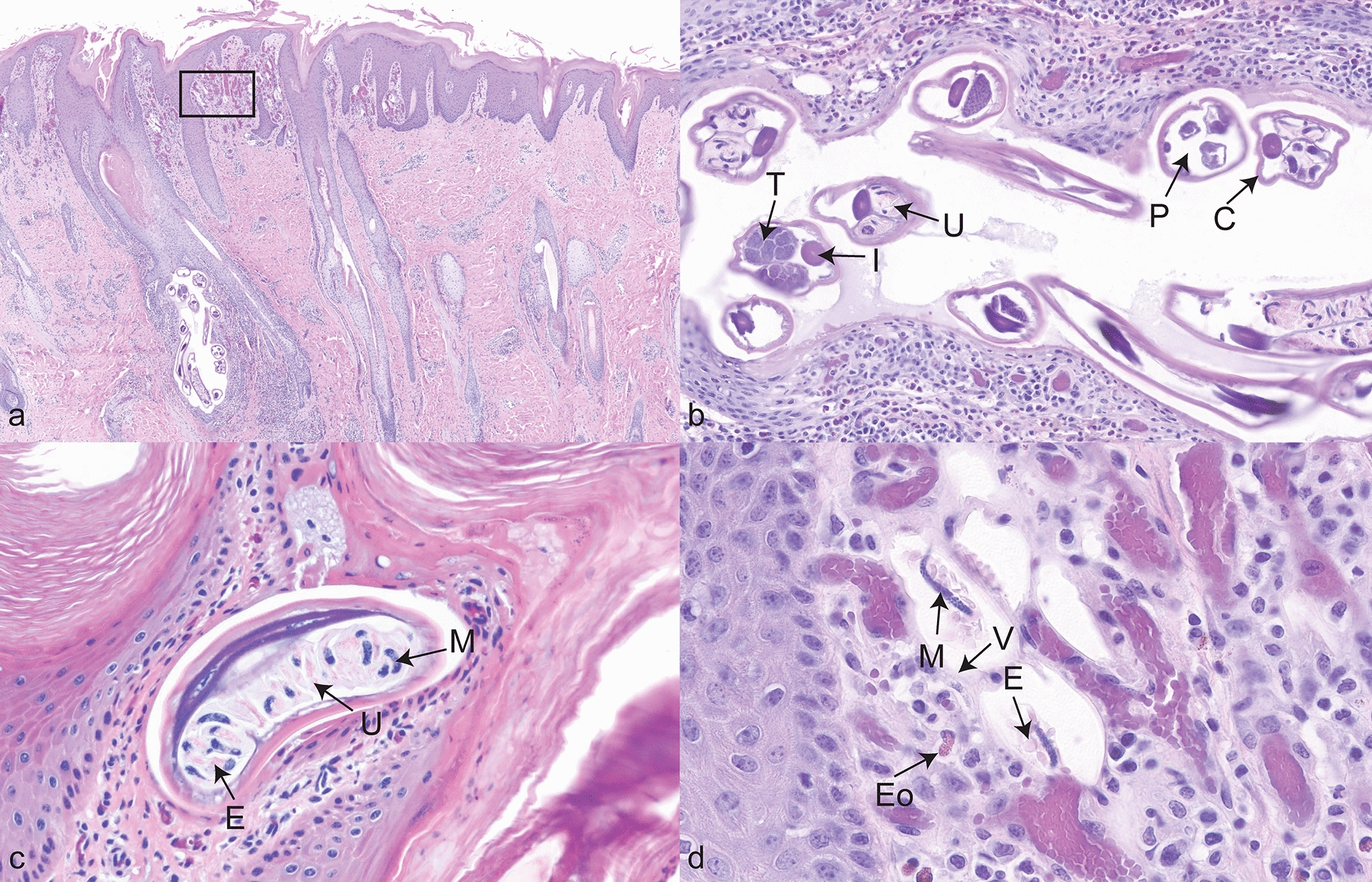
Characteristic histological lesions of *S. stilesi* dermatitis. **a** Severe, chronic, multifocal eosinophilic dermatitis and folliculitis with marked epidermal hyperplasia, orthokeratosis, and intralesional adult nematodes and first-stage larvae (L1); 40× magnification. Black box, lower magnification of D; **b** multiple cross sections of adult nematodes. 200× magnification. T, testicular tissue; I, intestine; U, uterus; P, pseudocoelom; C, cuticle; **c** cross section of an adult nematode uterus; 400× magnification. U, uterus; M, L1; E, eosinophilic disc; **d** L1 with surrounding eosinophilic discs, entrapped within a vitelline membrane in the superficial dermis; 600× magnification. M, L1. V, vitelline membrane. E, eosinophilic disc; Eo, eosinophil

Histologically, the haired skin showed eosinophilic and neutrophilic perivascular dermatitis with furunculosis and folliculitis. The epidermis was markedly hyperplastic with rete peg formation, prominent intercellular bridging (spongiosis), and swollen keratinocytes (intracellular edema). The superficial layers often had serocellular crusts and intra-corneal pustules filled with neutrophils, eosinophils, bacterial colonies, and accompanying orthokeratotic and parakeratotic hyperkeratosis. Blood vessels within the superficial dermis were commonly dilated and congested, along with varying degrees of dermal edema and fibrosis. Of 22 cattle examined, 13 samples had cross sections of adult nematodes consistent with *S. stilesi*. Adults were often found in the superficial dermis, around 1–2 mm below the epidermis and identified within intact follicles, sebaceous ducts, crusts, and areas of furunculosis (Fig. 2a). Adult cross sections were approximately 300 × 150 µm and characterized by pseudocoelom, cuticle, coelomyarian musculature, intestinal tract, reproductive organ (uterus or testis), and lateral alae (Fig. 2b). The uterus often contained L1 (Fig. 2c).

Of the 13 cattle with adult *S. stilesi*, 5 cases had L1 within the superficial dermis with minimal inflammatory reaction. Specifically, the L1 were most identified within the dermal projections (dermal papillae) that interdigitate within the overlying rete pegs of the hyperplastic epidermis. L1 were 20–30 µm long and encysted in a 50-µm sacculus lined by 1-um-thick membrane and filled with distinct small eosinophilic discoid material (Fig. 2d). Skin samples without adult nematodes also had no L1. Out of the five cases in which L1 were observed, four were necropsied within December and January and one from mid-August.

### Transmission electron microscopy

**Fig. 3:**
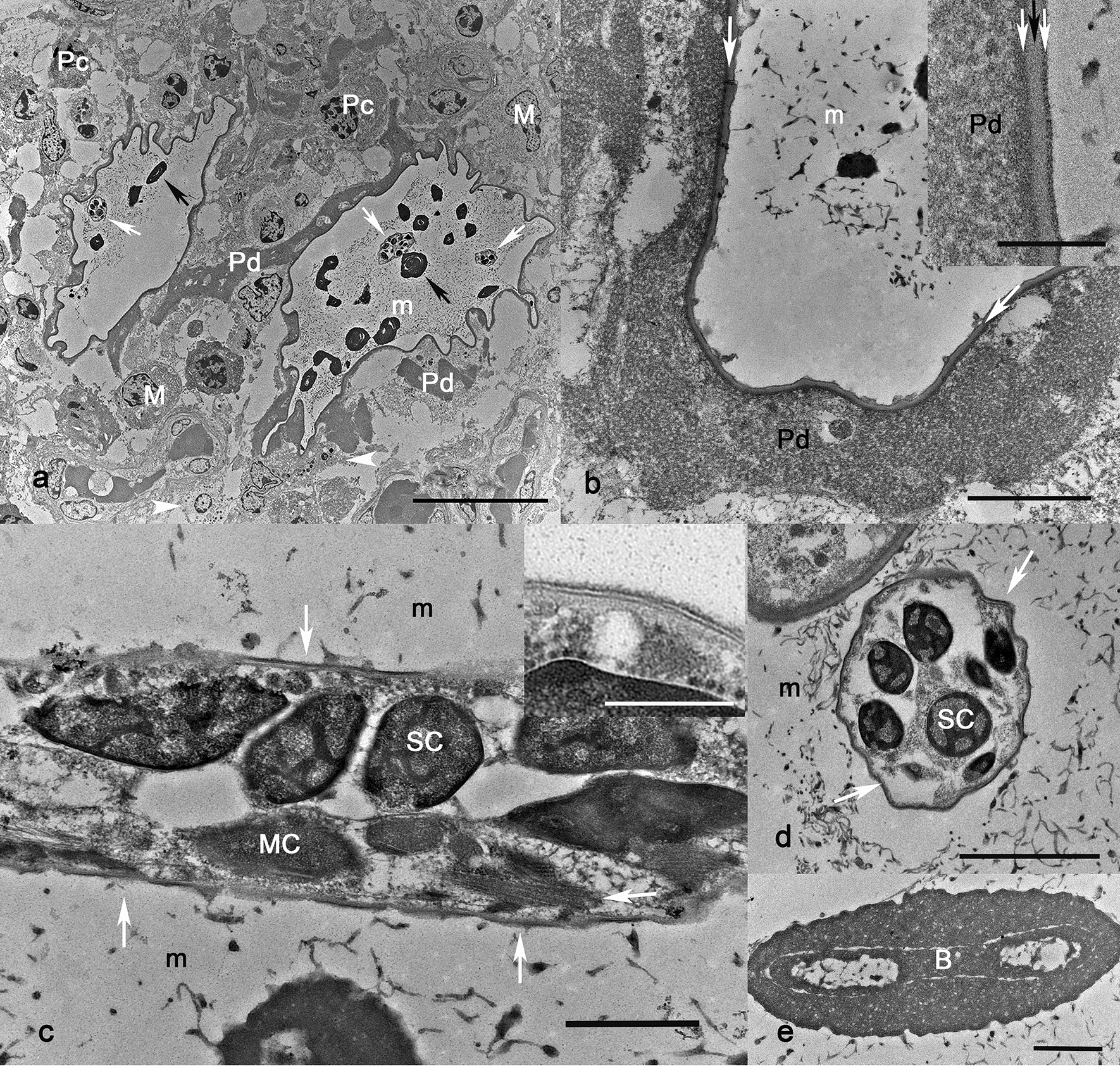
First-stage larvae (L1) and vitelline membrane of *Stephanofilaria stilesi*, bovine, skin. Transmission electron microscopy (TEM). **a** Two parasitic sacculi lined by vitelline membrane containing cross sections of larva (white arrow) and “eosinophilic disk” (black arrow) suspended in a matrix (m). Parasitic vitelline membrane and surrounding inflammation; proteinaceous material (Pd), plasma cells (Pc), macrophages (M), degranulated mast cells (arrowheads). Scale bar = 20.0 µm. **b** Vitelline membrane of the parasite (white arrows) and surrounding thick deposit of a granular/fibrillary material. Scale bar = 2.0 µm. Inset: magnification of the “vitelline membrane;” external and internal layers (white arrows), middle (layer black). Scale bar = 0.5 µm. **c** Sagittal section of a larva: cuticle (white arrows), somatic cells (SC), muscle cell (MC), parasitic matrix (m). Scale bar = 1.0 µm. Inset: magnification of the cuticle displaying its trilaminar structure. Scale bar = 0.25 µm. **d** Cross section of a L1 larva: cuticle (white arrows), somatic cells (SC), parasitic matrix (m). Scale bar = 2.0 µm. **e** “Eosinophilic disk” and elongated core (B) surrounded by electrode amorphous material. Scale bar = 0.5 µm

The vitelline membrane, a 50-µm sacculus containing a single L1 larva, was ultrastructurally composed of a trilayered membrane. The trilayered membrane was comprised of a 0.16-to-0.21-µm membrane externally surrounded by variable amounts, up to 3.32 µm, of amorphous electron-dense granular/fibrillary substance interpreted as deposition of host proteins (Fig. 3). The trilayered membrane was composed of an external and internal electron-dense material and a middle gray lamina. Each trilayered membrane contained sagittal and cross sections of a single L1 larva suspended in a matrix of filamentous electron dense substance (Fig. 3). L1 varied from 1.70 to 4.47 µm in diameter. A cuticle and somatic cells including muscle cells defined the L1 body. The cuticle was 0.5 to 0.6 µm and constituted of three layers. The individualized, amorphous, electron-dense discoid spheres, known as “eosinophilic discs” in histology, measured approximately 2.75 to 5.72 µm. These “eosinophilic discs” had a round to elongated (up to 1.07 to 4.5 µm in length) core wrapped with multiple laminated layers of fibrillary/granular substance.

### Molecular characterization

Four out of eight tested sets of primer successfully amplified *S. stilesi* DNA (Table 1), including the mitochondrial *cox1* (~ 680 bp) and 12 s (~ 450 bp) and 2 ribosomal, 18S rDNA (~ 730 bp) and 28S rDNA (~ 850 bp), whose sequences were generated by Sanger sequencing. To our knowledge, these were the first sequences available for *S. stilesi*.

### Tissue sample screening

Overall, the PCR targeting the *cox1* region was successful in detecting *S. stilesi* DNA in 6 out of the 22 cattle cases (27%) (Table 2). Of the 13 cases with either adults or L1 present, 5 produced a sequence matching the newly identified *S. stilesi* sequence (38%). In contrast, of the nine cases without either adult worms or L1, only a single sample produced a sequence (11%). Furthermore, triplicates produced an additional two *S. stilesi* sequences in cases with either adults or L1 present (7/39; 18%) but no more additional sequences when both were absent (1/27; 4%).

### Phylogenetics and pairwise distance

**Fig. 4:**
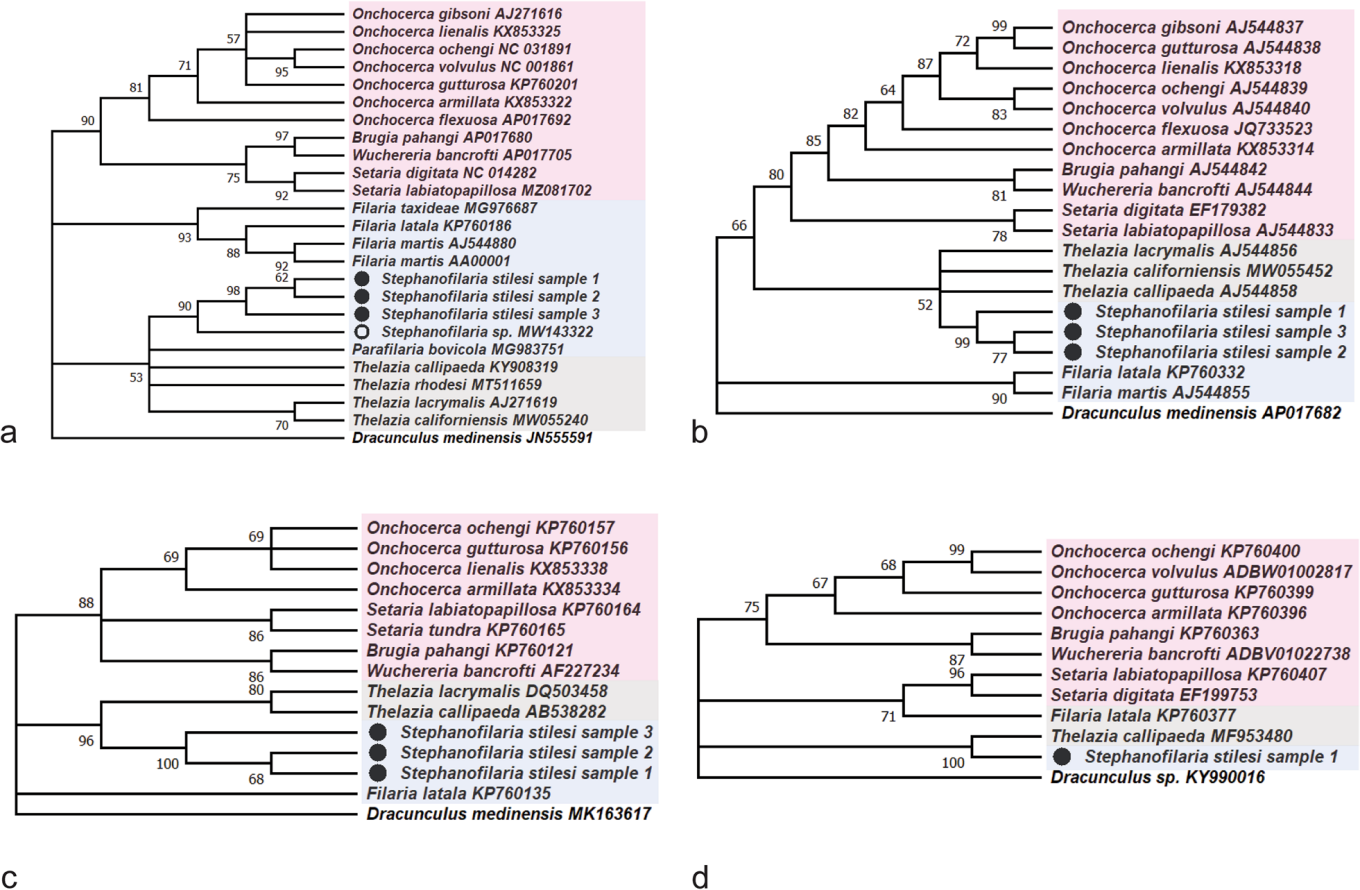
Maximum Likelihood tree depicting phylogenetic relationship of the *cox1* (681 bp), 12S (616 bp), 18S (715 bp), and 28S (1081 bp) genes between various known Filarioidea species, including the first sequence of *Stephanofilaria stilesi*, created with MEGAX. **a** *cox1* gene. **b** 12S gene. **c** 18S gene. **d** 28S gene. Branches with < 50% bootstrap support were collapsed. To show comparisons, *Stephanofilaria* sp. from Naseem et al. [47] is labeled with an unfilled circle, and *S. stilesi* samples are labeled with a filled circle. In addition, the families are portrayed in different colors to highlight species of Onchocercidae (pink), Thelazioidea (gray), and Filariidae (blue). Each *S. stilesi* sample has been accessioned in GenBank (COI: OP589131-3; 12S: OP596210-12; 18S: OP596207-9; 28S: OP596263)

Three individual sequences originating from DNA extracted from cattle skin containing *S. stilesi* L1were generated using *cox1* primers, separate from those of the molecular screening, and deposited at GenBank (accession nos. OP589131-3). All samples were trimmed according to the *cox1* length of the *S. stilesi* samples (681 bp). Phylogenetic analysis revealed that *S. stilesi* is closely related to the unnamed *Stephanofilaria* sp. from cattle in Australia, with 88% bootstrap support (Fig. 4). Pairwise analysis (Table 3) showed low intraspecies variation among all three *S. stilesi* samples at 99.85–100.00% (681/681 bp) and a genetic distance between 89.99 and 90.26% (435/681 bp) between *S. stilesi* and the *Stephanofilaria* species from Australian cattle [47]. No other parasite species clusters in the same monophyletic clade as the *Stephanofilaria* samples, even though other species currently allocated within Filariidae were represented within the initial phylogenetic analysis, namely *Parafilaria bovicola* Tubangui, 1934, *Filaria latala* Chabaud & Mohammad, 1989, *Filaria martis* Gmelin, 1790, and *Filaria taxideae* Kleppner, 1969. Pairwise comparisons of *S. stilesi* to other members within Filariidae were similar to those between Filariidae and Onchocercidae. Additional *S. stilesi* sequences were generated by using 12S (accession nos. OP596210-12), 18S (accession nos. OP596207-9), and 28S (accession nos. OP596263) primers. Phylogenetic trees constructed from these molecular markers revealed similar results to *cox1* analysis (Fig. 4).

**Table 3:** Average pairwise comparisons (similarity) of *cox1* gene with different Filarioidea species and *Thelazia californiensis*, with ranges in parentheses

Parasite Species	*Stephanofilaria stilesi* samples	*Stephanofilaria* sp.	*Parafilaria bovicola*	*Filaria taxidae*	*Onchocerca gutturosa*	*Thelazia californiensis*	References
Accession #	OP589131-3	MW143322	MG983751	MG976687	KP760201	MW055240	
*Stephanofilaria stilesi*	99.90%						Present study
	(100.00–99.85%)						
*Stephanofilaria* sp.	90.17%	–					Naseem et al. [47]
	(89.99–90.26%)						
*Parafilaria bovicola*	75.47%	72.95%	–				Oehm et al. [48]
	(75.31–75.54%)						
*Filaria taxideae*	39.03%	54.29%	55.73%	–			Mulreany et al. [49]
	(39.15–39.80%)						
*Onchocerca gutturosa*	81.87%	73.81%	82.35%	52.38%	–		Lefoulon et al. [45]
	(81.69–81.95%)						
*Thelazia californiensis*	77.34%	74.71%	77.73%	53.37%	79.45%	–	Sobotyk et al. [50]
	(77.15–77.43%)						

## Discussion

Although a common dermatitis of cattle in North America, much remains undescribed about the histological features of *S. stilesi*, and very little is known about aspects of its evolutionary biology and taxonomic classification. This can be partly attributed to the dated literature on stephanofilariasis in North American cattle [17]. However, more modern histological techniques have been recently applied to dermatological lesions caused by another *Stephanofilaria* species in cattle of Australia [15]. Our study is the first to our knowledge to use TEM for L1 visualization within *S. stilesi*. TEM was able to evaluate the composition of the “vitelline membrane,” which reveals a trilayered membrane. Despite its veterinary relevance, *S. stilesi* has, to our knowledge, never been genetically sequenced until this present study. In addition to its diagnostic utility, molecular data can advance the knowledge on the biology and epidemiology of different *Stephanofilaria* species, including defining their host and vector associations, as well as elucidating their evolutionary relationships and taxonomic classification. However, currently, there is a scarce amount of genetic data for definitive comparisons among nematodes, especially in the family Filariidae [22]. This study highlights and enhances some of the former *S. stilesi* studies and the need to explore this overlooked parasitic infection with more modern scientific techniques.

### Stephanofilariasis importance among ungulates

Apart from *S. stilesi* subclinical and clinical impacts on domestic North American cattle, many *Stephanofilaria* species are of medical importance to a variety of domestic and wild ungulate hosts worldwide. In Indonesia, *Stephanofilaria dedoesi* Ihle & Ihle-Landenberg, 1933, causes dermatitis on the ears, neck, head, dewlap, and sternum of cattle, known as “cascado” [19]. In Malaysia, *Stephanofilaria kaeli* Buckley, 1937, causes dermatitis on the feet, ears, and teats of cattle [23]. In India, *Stephanofilaria zaheeri* Singh, 1958, causes dermatitis in the ears of buffaloes, and *Stephanofilaria assamensis* Pande, 1936, causes dermatitis on the hump of Zebu cattle, known as hump sores [7, 24, 25]. There has been, however, some debate regarding the synonymy of these two species. In Japan, *Stephanofilaria okinawaensis* Ueno & Chibana, 1977, was described and has been associated with dermatitis in the muzzles and teats of cattle [26], but the validity of the species was later questioned by taxonomists [11].

More recently, giraffe skin disease (GSD), an ulcerative dermatitis in giraffes, has been linked to parasitic nematodes suspected to belong to the genus *Stephanofilaria*. Histological lesions, found on the neck, chest, axilla, and shoulders, reveal intralesional nematodes similar to stephanofilariasis, and molecular testing revealed that these organisms were most closely related to other filarioid organisms using 18S primers, in the absence of direct comparison to *Stephanofilaria* sequences at time of publication [13]. Pairwise data in our study showed *S. stilesi* to have an average similarity of 97.05% (97.16–96.83%) to the nematodes present in GSD. While not indicative of a conspecific relationship to *S. stilesi*, due to the extremely conserved nature of the 18S genetic region, it is possible that these parasites may belong to the genus *Stephanofilaria* or its subfamily. Characterization of additional markers of the nematode involved in GSD would be useful to further elucidate its identity and relationship with *S. stilesi*.

*Stephanofilaria* species causing skin lesions have also been relatively well documented in other ungulate hosts [10–14]. In pigs from the current Democratic Republic of Congo, *Stephanofilaria boomkeri* Bain, Van der Lugt & Kazadi, 1996, has been described as causing ulcerative dermatitis along the teats, ears, and legs with accompanying epidermal hyperplasia and intralesional nematode worms [10]. In black rhinoceroses, *Stephanofilaria dinniki* Round, 196, was described as causing otitis and erosive and ulcerative dermatitis on the flank behind the elbow. Lesions ranged between 5 and 20 cm and revealed similar findings of epidermal hyperplasia and intralesional nematodes [12]. In the hippopotamus, *Stephanofilaria thelazioides* Boomker, Bain, Chabaud & Kriek, 1995, has been described as causing 5-cm-diameter ulcerative dermatitis in the shoulder [14]. Overall, *S. stilesi* infections tend to vary in location and severity and are more widely distributed compared to the North American *S. stilesi*. Considering their relevance to the veterinary, wildlife health, and conservation communities, there is a significant need to better understand the evolution, biogeography, and ecology of these *Stephanofilaria* species.

### Stephanofilariasis gross pathology, histopathology, and transmission electron microscopy

The macroscopic lesions of variable size identified in our cases were similar to previously described reports of stephanofilariasis in North America, Australia, Africa, and Europe [1, 8, 10, 19]. While *S. stilesi* lesions seem to always occur in the ventral midline of cattle, as confirmed in our study, other *Stephanofilaria* species occur in different anatomic locations in the skin of cattle, such as the medial canthus of the eye, ear, neck, withers, shoulder, hump, dewlap, dorsum, ventral abdomen, teats, and scrotum [8, 9, 15, 19]. The age of cattle in our study ranged between 8 months and 12 years. Although our study was not designed to assess age association and severity of lesions, it seems that older animals tend to have larger lesions compared to those seen in younger animals. The larger lesions are likely correlated with prolonged exposure, as older animals with larger lesions often had extensive fibrosis, a common indicator of chronicity. A greater number of females were identified in this study, which is likely due to a bias in animals received for necropsy during the limited sampling period. No correlation could be made between the time of year and severity of skin lesions.

Skin lesions due to *S. stilesi* are prominently found on cattle in rangeland and irrigated pastures because such environments are ideal for horn flies. Probably, the main predisposing factor for *S. stilesi* in cattle is the presence of horn flies in the area. In Texas, horn fly populations tend to drop in midsummer and winter. The peak of horn fly populations is in early fall or spring [27]. Lesions in cattle are not usually identified until 1 year of age and often start as an exudative, crusted, and bloody area of the skin [1, 9]. The age of onset is likely associated with a combination of factors, including calving season variation among producers, exposure variability due to housing/environmental conditions/horn fly populations, and incubation time for both nematodes and development of gross lesions. Over time, the skin slowly heals and becomes smooth, dry, and lichenified; this is histologically characterized by marked dermal fibrosis and lack of inflammation. Cattle can recover fully from *S. stilesi* lesions as they age, with many cattle of 15–20 years old having no lesions [1, 9].

Epidermal and dermal histological lesions associated with stephanofilariasis are rarely described, but our findings are consistent with one study [8] describing chronic eosinophilic folliculitis with hyperkeratosis and parakeratosis. In our examinations, the severity of folliculitis, dermatitis, and furunculosis varied from mild to marked and often corresponded with the chronicity of the lesion. The more acute dermatitis often exhibited marked inflammation and edema, while chronic lesions often exhibited dermal fibrosis and minimal inflammation. Additionally, more acute to subacute dermatitis with thin crusts tended to have more adult nematodes and L1. In addition to the inflammatory response associated with the nematode, horn fly bites may also elicit an inflammatory response on their own, which has been associated with chronic lymphoplasmacytic, eosinophilic, and neutrophilic dermatitis with marked fibrosis [2, 28, 29]. These lesions show significant similarities to the chronic cases within this study. The evidence of an eosinophilic infiltrate without the nematode identification may imply an ectoparasitic infestation (i.e. demodicosis, chorioptic mange) or hypersensitivity reaction due to the fly bite. The gross and histological findings may be due to a combination of *S. stilesi* infection and horn fly infestation, and the vector may contribute to the chronicity and crust formation even when nematodes are absent on histology [28, 29].

Histological description of the adult nematode and L1 is consistent with what has been previously described in the scientific literature [6, 8, 15, 30]. Although not often emphasized, it is important to note that nematodes are often located within the epidermis superficial dermis, particularly within the first 4 mm of skin. Another diagnostic feature of stephanofilariasis is identifiable L1 within the uteri of females, since *Stephanofilaria* spp. are viviparous and have larva instead of shelled ova [1].

The presence of L1 was always accompanied by adult nematodes and most commonly found between the months of December and January. This finding may imply that the L1 are most commonly found the winter season, which is generally mild in the region of Texas from which samples originated. Microfilariae of many filarioid nematodes are hypothesized to create an inflammatory response through endosymbiont bacteria, like *Wolbachia* within *Dirofilaria immitis* and *Brugia malayi* [31, 32]. However, no such bacteria could be identified on TEM in the present study.

Besides other common differential diagnoses of cattle skin lesions such as trauma, dermatophilosis, and fungal/bacterial dermatitis, another possible parasitological cause is *P. bovicola*, which has not been reported in North America [33]*.* This parasite seems to be the closest related Filariidae species to *S. stilesi* outside of the genus *Stephanofilaria* and has an average similarity of 75.47% (75.31–75.54%) to *S. stilesi* (649/681 bp) (Table 3). *Parafilaria bovicola* is transmitted by non-biting flies, *Musca lusoria* or *Musca xanthomelas*, and commonly found in parts of Asia, Africa, and Europe, including the UK and Germany [34, 35]. Parafilariasis infection tends to produce painful subcutaneous nodules usually identified within the shoulder, axilla, neck, lateral thorax, and lateral abdomen [33, 35]; therefore, location of lesions can be relevant for differential diagnosis for *S. stilesi* infection. On histopathology, a marked eosinophilic perivascular dermatitis and panniculitis with intralesional nematodes is identified. In contrast to stephanofilariasis, previous reports indicate that inflammation is much deeper in the dermis and subcutis, with no mention of folliculitis or furunculosis [33].

Our TEM findings on *S. stilesi* indicate that the anatomy of the L1 is similar to what has been described for Thelazioidea and Filarioidea nematodes, with a trilaminate cuticle, somatic cells, and myofibers [36]. To our knowledge, this is the first time that the “vitelline membrane” and “eosinophilic discs” have been evaluated on TEM. The “vitelline membrane” surrounding the L1 is a trilayered membrane, which has partial structural similarities to what has been described in the eggshell of *Caenorhabditis elegans*, a nematode within the family Rhabditidae [37]. The eggshell within *C. elegans* is composed of six layers, the outermost vitelline layer, chitin layer, chondroitin proteoglycan (CPG) layer, extra-embryonic matrix (EEM) layer, permeability barrier layer (also known as vitelline membrane previously), and peri-embryonic layer. The three outer layers (vitelline layer, chitin layer, and CPG layer), measuring approximately 300–400 µm thick, comprise what is commonly recognized as the outer eggshell seen on light microscopy and EM [38]. The vitelline layer is a thin membrane present on the oocyte and is maintained post-fertilization. The chitin layer is an aminopolysaccharide polymer that gives structure and shape to the shell of the egg. The CPG layer is composed of proteoglycans and acts as a barrier.

The three inner layers (EEM, permeability layer, and peri-embryonic layer) are very thin layers that surround and protect the larvae and are not commonly seen on light microscopy. The EEM layer is a fluid-filled layer that separates the outer trilaminar outer shell from the inner embryo. The permeability barrier layer (commonly called the vitelline membrane) and the peri-embryonic layers allow for osmotic integrity of the embryo [39]. They serve as a barrier from the external environment when the outer eggshell (vitelline layer, chitin layer, and CPG layer) is lost [37]. In the case of *S. stilesi* larvae, there is no clear outer eggshell structure, but the trilayered membrane, commonly referred to as vitelline membrane, structurally resembles a portion of the eggshell. The term vitelline membrane, or what is now known as the permeability barrier layer, can cause confusion because of its similar name with the vitelline layer of the outer eggshell. Therefore, the term vitelline membrane may not accurately represent the membrane surrounding the larvae because the permeability barrier layer does not resemble the membrane we have identified on TEM. The origin of this membrane is unclear, but it is likely created within the reproductive tract of the female nematode. More research needs to be performed to understand the formation of this membrane and its relation to an eggshell from oviparous nematodes.

### Integrating molecular diagnosis to gross examination and histopathology

Overall, diagnosis of active *Stephanofilaria* infection appears most reliable when combining molecular testing and histopathological findings. While initial PCR screening may not be ideal for a diagnosis by itself, this study suggests that PCR may aid in a confirmatory diagnosis when histopathology reveals no evidence of nematodes and vice versa. In addition, PCR has the advantage of discerning different species of *Stephanofilaria*, which can influence management of domestic and wild ungulates without laborious microscopic techniques. A study from Australia revealed an overall sensitivity of 71.4% with their molecular assays and 32% through nematode identification on histopathology. Samples were from hides of freshly slaughtered animals, and nematodes were extracted using the saline recovery technique. Our molecular assays had a lower sensitivity rate (27%), and the difference in this case is attributed to multiple factors, including timing of sample collection from dead cattle submitted to the necropsy service and sample freezing before PCR analysis. In our study, skin samples that were submitted to DNA extraction were acquired at sites adjacent to histopathological examination, without prior confirmation of the presence of *S. stilesi* adult or larval stages. These differences in sample collection, handling, and processing may have led to a lower overall sensitivity of our molecular assays.

### *Stephanofilaria* taxonomy and evolutionary history

To our knowledge, this is the first instance of *S. stilesi* being genetically sequenced in North America. The only genetic information for an unnamed species of *Stephanofilaria* became recently available from a study in Australia [47]. Phylogenetic analysis of a fragment of the *cox1* gene reveals a close relationship between both *Stephanofilaria* species, which support taxonomic relatedness (~ 90.17%) (Table 3). However, *S. stilesi* and the Australian cattle *Stephanofilaria* appear distantly related to other species within Filariidae, in which it has been historically allocated (Fig. 4). While morphological and ecological similarities have influenced the allocation of the genus *Stephanofilaria* within Filariidae and Filarioidea, early researchers have suggested this allocation was provisional and highlighted similarities with the genus *Thelazia* and Thelazioidea, including aspects of the morphology of the larval stages, as discussed above [30–32]. Recent genetic evidence showed that the Australian cattle *Stephanofilaria* sp. was phylogenetically closer to *Thelazia* species, rather than to species within *Filaria* [47]. In fact, our cox1 findings further support the closer relationships with *Thelazia* and *P. bovicola,* which has also been placed within Filariidae. The additional genetic markers utilized in this study (e.g. 12S, 18S, and 28S) revealed similar results on the closer association of *Stephanofilaria* and *Thelazia* and others compared to species of the genus *Filaria* (Fig. 4). If these findings are confirmed through more robust genetic information, the subfamily Stephanofilariinae, with at least the genera *Stephanofilaria* and *Parafilaria*, and species within, would have to be reallocated from the Filarioidea to Thelazioidea. Decisions on its allocation to sub-family level within Thelaziidae or family level within Thelazioidea remain to be resolved. Nevertheless, this could have direct implications for our findings. Following the current evidence from our study and the recent literature, it seemed erroneous to refer to the initial larval stage of *S. stilesi* as “microfilariae;” therefore, we opted to refer to these as L1 [40–43].

### Limitations of this study

The histological evidence of adult nematodes (13/22) and molecular screening using the *cox1* PCR (Table 3) (6/22) are variable. This could be attributed to the specific tissue section that was taken compared to the often large lesion associated with the infection. If the sample section does not contain any nematode cross section, then it will not be able to detect the presence of *Stephanofilaria* on PCR or histology. This could be attributed to the specific tissue section that was taken compared to the often large lesion associated with the infection. If the sample section does not contain any nematode cross section, then it will not be able to detect the presence of *Stephanofilaria* on PCR or histology. In addition, samples were collected from animals up to 48 h after death, which would lower DNA viability due to some level of autolysis. The saline recovery technique was not used in our study because of the prolonged postmortem interval in some specimens, but this technique may increase the chance of nematode recovery from skin lesions [47]. Furthermore, our histological findings reveal that more chronic lesions (marked dermal fibrosis with absence of folliculitis and furunculosis and less severe inflammation) had fewer to no nematodes within section. It is possible that these lesions healed over with no ongoing *S. stilesi* infection. In the absence of nematodes within acute lesions, the same gross changes of well-demarcated, crusting dermatitis surrounding the umbilicus and histological findings of eosinophilic or neutrophilic dermatitis and folliculitis, furunculosis, and hyperkeratosis lead us to believe that *S. stilesi* was the cause for these lesions. As additional genetic data become available for *S. stilesi* and other species within the genus, additional markers could be proven to be of diagnostic value, and more sensitive assays and technologies could be developed and employed.

It is important to note that the cattle from this study are only from Texas and may not reflect the same pathology from other infected cattle populations in North America, assumed to be possibly affected by *S. stilesi*, or worldwide, where various *Stephanofilaria* species are present and known to cause lesions of variable severity in different anatomic locations of cattle. Additionally, most cattle identified were beef breeds, particularly Hereford, Brangus, and Black Angus, which may or may not be a good representation for lesions identified within all cattle breeds. It is also unknown whether the limited sample size could have any effect. For example, an insufficient number of cases could have biased correlation between adult/L1 identification in sex and seasonality. Furthermore, suspicion of stephanofilariasis infection is dependent on lesion recognition.

## Conclusions

The purpose of this study was to better understand the gross and histological pathology associated with *S. stilesi* and characterize their respective molecular markers to enhance the ability for diagnosing bovine stephanofilariasis in North America. To fill these knowledge gaps, we utilized multiple techniques, including histology, TEM, and molecular tools to improve *Stephanofilaria* diagnosis. Overall, the ability of PCR or histopathology to provide evidence of *S. stilesi* presence in the absence of either in our study highlights the advantage of using both rather than a single technique for stephanofilariasis diagnosis. In addition, the TEM analysis of *S. stilesi* reveals that the vitelline membrane is composed of a trilayered membrane. Phylogenetic evidence provided in our study supports previous hypotheses of a closer relationship of *Stephanofilaria* and Stephanofilariinae, with Thelazioidea, which emphasizes the need for reassessing their current taxonomic with possible reallocation from Filariidae and Filarioidea to the former taxon.

## Data Availability

The data will be made available upon request to the corresponding author.

## Abbreviations

PCR: Polymerase chain reaction
TEM: Transmission electron microscopy
GSD: Giraffe skin disease
TVMDL: Texas A&M Veterinary Medical Diagnostic Laboratory
